# Demographics and Mortality Rates of Surgical Emergencies Treated at the Casualty Operation Theater: A Six-month Retrospective Analysis

**DOI:** 10.7759/cureus.7658

**Published:** 2020-04-13

**Authors:** Salman Jafferi, Ghina Awais, Rubiya Naeem, Jabbar Ghufran Syed, Muhammad Ali, Hamra Afridi, M Hassan, Shahid Rasul

**Affiliations:** 1 Surgery, Jinnah Postgraduate Medical Centre, Karachi, PAK; 2 General Surgery, The Indus Hospital, Karachi, PAK; 3 Cardiology, Jinnah Postgraduate Medical Centre, Karachi, PAK; 4 General Surgery, Jinnah Postgraduate Medical Centre, Karachi, PAK; 5 General Surgery, Kulsoom Bai Valika Social Security Hospital, Karachi, PAK; 6 Surgery, Jinnah Postgraduate Medical Cente, Karachi, PAK; 7 Surgery, Jinnah Postgraduate Medical Centre, Jinnah Sindh Medical University, Karachi, PAK

**Keywords:** demograhics, mortality rates, casualty, operation theater, surgical emergency

## Abstract

Introduction

Emergencies such as appendicitis, peritonitis, road traffic accidents and gunshots require immediate surgical intervention. Patients are first resuscitated at the emergency department and then shifted to the casualty operation theater (COT). COT is a state-of-the-art operation theater that is open 24/7 and ready to deal with any surgical crisis. Once surgery is performed, the patients are admitted to the surgical ward for post-operative care.

Jinnah Postgraduate Medical Centre (JPMC) is the largest tertiary care hospital in Karachi. There is very limited data on the cases that are dealt with on regular basis at the COT in JPMC. Here we break the mold and analyze the various aspects of surgical emergencies treated at the COT over the course of last six months.

Objectives

To evaluate the demographics and mortality rates of emergencies treated at the COT in the last six months.

Methods

This was a retrospective study, held for six months (July 1^st^ 2019 to December 31^st^ 2019). Data was obtained from the Records and Administration section, Surgical Unit IV (ward 21), Jinnah Postgraduate Medical Centre.

Results

Three hundred and fifty-five patients were inducted into the study, predominantly male. Majority (71.54%) of the referrals were made from within the city. The mean age of the patients was 48.57 ± 14.92 years. Appendicitis was the most common emergency treated at the COT. The overall mortality rate was 23.94%. Peritonitis and road traffic accidents contributed significantly to the mortality rate.

Conclusion

Surgical emergencies treated at the COT have a high mortality rate at one week. Prompt recognition, early referrals and intervention can help reduce mortality in the future.

## Introduction

Surgical emergencies make up only a fraction of the total cases seen at the emergency department. However, almost all of these require immediate intervention [1]. This includes resuscitation, lab work up, coordination with the anesthesiology team, pain control, ordering relevant radiological investigations and most importantly time management with respect to the signs and symptoms. Unlike other scenarios a conservative approach is not warranted [2].

Cases of peritonitis, intestinal obstruction and road traffic accidents (RTAs) among others are best treated at the casualty operation theater (COT). The COT is a 24/7 service that has the expertise and equipment to deal with any and all surgical emergencies. The COT however is limited by the number of operation theaters available at a given time.

The COT at Jinnah Postgraduate Medical Centre (JPMC) sees an unusually high number of cases each day; it is the most referred centre in all of Sindh. Unfortunately data with regards to type of surgical case, patient characteristics and mortality is all but absent to the best of our knowledge. Here we indulge in a retrospective analysis of the demographics and outcomes of patients treated at the COT in the last six months.

## Materials and methods

Study design

This was a retrospective study.

Data source

All data was collected from the Records and Administration section of Surgical Unit IV (ward 21), Jinnah Postgraduate Medical Centre, Karachi. Patient confidentiality was made certain. Patients were only referred to by their given numbers.

Duration

This study was held for a period of six months from July 1st 2019 to December 31st 2019.

Inclusion criteria

Male or female patients aged 18 years or older that presented at the emergency department requiring urgent surgical intervention were eligible for induction into this study. All patients were first resuscitated at the emergency department, once hemodynamically stable the patients were shifted to the COT. Relevant surgical intervention(s) were performed at the COT.

Patients were then admitted to ward for post-operative care. All data was maintained and stored at the Records and Administration section. Outcomes such as discharge, expiry, and discharge on request or leaving against medical advice (LAMA) were recorded as well. This data was retrospectively analyzed for the purposes of this study. Patient confidentiality was made certain.

Note: Surgical unit IV (ward 21) conducts two 24-hour emergency calls every week, on Thursdays and Saturdays only. Therefore data is only recorded for these two days.

Exclusion criteria

Patients receiving chemotherapy or radiotherapy, patients with previous surgeries and patients requiring a multi-disciplinary approach were all excluded from the study. Burn victims were also not included in this study.

Sample technique

Non-probability and consecutive sampling was used for this study.

Data analysis

Statistical package for social sciences (SPSS) version 21 (IBM Corp, Armonk, NY) was employed to analyze the data.

Primary outcome

Post-operative survival at one week was the primary outcome evaluated in this study. Overall mortality and mortality for each specific emergency was also analyzed.

## Results

Three hundred and fifty-five patients were inducted into the study. The mean age of the patients was 48.57 ± 14.92 years and almost 60% of the patients were male. Majority of the females were housewives and this was also the most common occupation. Referrals were predominantly made from within the city. Demographics are shown in Table 1.

**Table 1:** Demographics of the patients inducted in this study.

	N (%)
Gender	
Male	206 (58.02%)
Female	149 (41.97%)
Residence	
Karachi	254 (71.54%)
Non-Karachi	101 (28.45%)
Occupation	
Housewife	98 (27.60%)
Laborer	67 (18.87%)
Unemployed	61 (17.18%)
Mechanic	32 (9.01%)
Shopkeeper	30 (8.45%)
Tailor	19 (5.35%)
Student	14 (3.94%)
Driver	11 (3.09%)
Teacher	10 (2.81%)
Businessman	7 (1.97%)
Security Guard	3 (0.84%)
Police officer	1 (0.28%)
Doctor	1 (0.28%)
Electrician	1 (0.28%)
Age (mean ± standard deviation)	48.57 ± 14.92 years

Appendicitis was the most frequent surgical emergency seen during the study period. Surgical emergencies treated at the COT are summarized in Table 2. One-week mortality was 23.94%. The highest number of deaths was seen with perforated viscera or peritonitis. However, road traffic accidents (RTAs) carried the highest in group mortality rate at 61.36%. Overall mortality and relative mortality within a group are summarized in Table 3.

**Table 2:** Surgical emergencies treated at the Casualty Operation Theater.

	N (%)
Appendicitis	89 (25.07%)
Perforated viscera/peritonitis	73 (20.56%)
Intestinal Obstruction	59 (16.61%)
Superficial Abscess	50 (14.08%)
Road Traffic Accident	44 (12.39%)
Strangulated hernia	19 (5.35%)
Gunshot wounds	9 (2.35%)
Intra-abdominal abscess	6 (1.69%)
Blunt trauma / Assault	6 (1.69%)

**Table 3:** Overall and relative mortality rates.

	One-week Mortality N (%)	Relative mortality for the specific emergency (%)
Perforated Viscera / Peritonitis	35 (9.85%)	47.94%
Road Traffic Accident	27 (7.60%)	61.36%
Intestinal Obstruction	12 (3.38%)	20.33%
Gunshot wounds	5 (1.40%)	55.55%
Strangulated Hernia	3 (0.84%)	15.78%
Appendicitis	2 (0.56%)	2.24%
Blunt trauma / Assault	1 (0.28%)	16.66%
Superficial Abscess	Nil	Nil
Intra-abdominal abscess	Nil	Nil
Overall mortality	85 (23.94%)	

## Discussion

The emergency department of Jinnah Postgraduate Medical centre receives approximately 1200 patients every day. Less than 5% of these patients require surgical intervention; this includes orthopedic and neurosurgical emergencies. On average the COT treats anywhere between 10 to 15 patients every day. This corresponds with the total number of patients analyzed in this study. Remember that data was recorded for two days only over the 26-week study period (see methods).

The COT is limited by the number of operating rooms, availability of experienced surgeons or anesthesiologists, high number of referrals, unavailability of blood products and reluctance of attendants to give consent. Assessment of labs and clearance for general anesthesia fitness only adds to the preoperative time [3]. The mix bag of aforementioned factors at times delay cases so much so that they are carried over to the elective operation theaters. This reduces the number of cases treated at the COT.

As with previous data in Pakistan most of the patients were males, but this did not bear any statistical significance; the mean age of patients was also consistent with previous reports [4]. More than a quarter of the patients were referred from outside of Karachi. It has been previously well documented that JPMC entertains a vast majority of cases from all over the province of Sindh [5]. This trend follows at the COT and adds to an ever-growing burden.

People from all walks of life were treated at the COT. Females were predominantly housewives or unemployed. Men had more varied professions. Most of the patients belonged to the “lower socioeconomic” spectrum of the workforce, as evident by their occupations. These are complex issues. We will refrain from social commentary and discussions as it is beyond the scope of this study.

Overall mortality was 23.94%, a relatively high rate. The main contributors to this high number were peritonitis and RTAs. These two pathologies had disproportionately high fatalities, which has somewhat construed the overall mortality rate. These are discussed in more detail later on. For all other emergencies the mortality rates were low or their numbers were too small to have a significant impact.

Appendicitis was the most frequent surgical emergency encountered in this study. This fact was previously reported by Jawaid et al., but with a much lower incidence [6]. Appendicitis usually has a benign course and good surgical outcome unless complicated by perforation. All deaths in this group were due to perforated appendicitis in this analysis.

Peritonitis is the bane of emergency departments all over the world. It is associated with many preoperative risk factors that contribute to a high mortality rate [7]. The preoperative risk factors must be recognized early on to improve outcomes. Development of sepsis, anemia, acute renal injury or failure, hypoalbuminemia and hypovolumemia are just some of the amenable preoperative risk factors.

Peritonitis was the second most common primary diagnosis recorded in this study. It had the highest overall mortality rate of 9.85%. As discussed above the cases of peritonitis are already complicated by several preoperative risk factors, the situation is made worse by late referrals and inadequate facilities at the primary and secondary care levels. It is the opinion of the authors that early recognition in spite of complications and co-morbidity can reduce mortality rates substantially.

The incidence of intestinal obstruction and superficial abscess was almost similar but their mortality rates were worlds apart. Superficial abscesses are all but a benign pathology and are usually found in the breast tissue [8]. Patients usually presented with pain and mass effect; rarely were there any systemic manifestations. A simple incision and drainage is required to cure it. If there were signs of sepsis prophylactic antibiotics were given, culture and sensitivity assays were carried out as well.

Intestinal obstruction on the other hand carried significant mortality. Increasing age (especially in the geriatric population) has been implicated with higher mortality rates [9]. The elderly also have a higher prevalence of comorbidity. Of the 12 patients that expired due to intestinal obstruction in this study, nine were 70 years or older. Three remaining cases were complicated by late presentation and shock.

Only 44 cases of RTAs were recorded in the last six months. This figure can be misleading. The actual cases of RTAs are much greater. Remember that cases that were referred to neurosurgery and/or orthopedics were excluded from this study; this is the case with many RTAs. Cases that purely involved the surgical team (without a multidisciplinary approach) were far and few between. Otherwise, approximately 30 cases of RTAs are seen every day at the emergency department.

Disturbingly high mortality rate was seen with RTAs. There are a couple of reasons for this. Most cases referred to JPMC are from intercity highways. Vehicles are traveling at speeds in excess of 100 miles per hour on these highways; furthermore most of the vehicles are heavy vehicles. These include trucks, buses and modified wagons. Heavy vehicle accidents have traditionally been associated with high mortality rates; similar rates were seen in our study [10].

All cases of strangulated hernia could have been easily avoided had the patients gone under elective repair. This apparent reluctance on part of patients and attendants remains unexplained and we cannot comment on it. Mortality rate of strangulated hernia was consistent with previous data. None of the patients in this study required a re-operation. Cases of peritonitis or bowel perforation were analyzed separately.

Data on all gunshot and trauma cases was difficult to retrieve as it was part of the medico legal system. The cause of death in these cases was major internal injury and bleeding. Yet again, the number of cases recorded is misleading. Many gunshot and trauma cases involve the neurosurgical and/or orthopedics department and almost always require a multidisciplinary approach. As such most cases were excluded from the study.

Shortcomings

This study was hampered by a few shortcomings. The effect of age and comorbidity on mortality rate was not analyzed. After the initial discharge patients were not followed up at all. This study did not take into account any post-operative complications that developed after the patients were discharged. Nutritional assessment before and after the surgery is always done in patients, but our study did not evaluate these parameters.

## Conclusions

All manner of surgical emergencies are treated at the COT from different parts of the province. Mortality rate for emergencies treated at the COT is high, mainly due to peritonitis and RTAs. Early recognition and prompt intervention at the primary or secondary levels can lead to better outcomes. A more comprehensive analysis in the future is needed to make recommendations.
